# A rare presentation of subcutaneous granuloma annulare: a case report of bilateral palmar and dorsal hand nodules with successful management

**DOI:** 10.1097/MS9.0000000000003642

**Published:** 2025-07-25

**Authors:** Zainab Srouji, Najem Salem, Rama Ghwich, Munir Mounla, Noura Abdul Rahman

**Affiliations:** a Faculty of Medicine, University of Aleppo, Aleppo, Syria; b Department of Dermatology and Venereology, Faculty of Medicine, University of Aleppo, Aleppo, Syria

**Keywords:** case report, corticosteroid, palms, pseudorheumatoid nodules, subcutaneous granuloma annulare

## Abstract

**Introduction and importance:**

Granuloma annulare (GA) is a benign, self-limited, chronic inflammatory dermatosis predominantly affecting children and young adults. Subcutaneous granuloma annulare (SGA) is a rare variant, chiefly observed in children, characterized by firm, asymptomatic nodules typically located on the lower extremities, buttocks, or scalp. Involvement of the palms or soles is exceptionally rare.

**Case presentation:**

We report the case of a healthy teenager presenting with bilateral subcutaneous nodules on both the palmar and dorsal surfaces of the hands. Histopathological examination confirmed the diagnosis of SGA. Given the patient’s aesthetic concerns, a treatment regimen of intralesional corticosteroid injections was initiated, leading to complete resolution of the lesions without recurrence during follow-up.

**Clinical discussion:**

This case represents the first documented instance of SGA involving both palmar and dorsal surfaces of the hands bilaterally in a healthy adolescent. While SGA is usually self-limited, cosmetic concerns or discomfort may necessitate therapeutic intervention. Intralesional corticosteroids have been employed with variable success in such cases.

**Conclusion:**

This case underscores the importance of considering SGA in the differential diagnosis of subcutaneous hand nodules in pediatric patients, even in atypical locations such as the palms and dorsal hands. It also highlights the efficacy of intralesional corticosteroid treatment in achieving satisfactory cosmetic outcomes without recurrence.

## Introduction

Granuloma annulare (GA) is a benign, self-limited, chronic inflammatory dermatosis that predominantly affects children and young adults, though it can occur at any age^[1]^. Several subtypes of GA have been described: localized, generalized, perforating, and deep or subcutaneous granuloma annulare (SGA)^[2]^.

SGA is a rare variant, predominantly seen in healthy children, characterized by firm, skin-colored to erythematous asymptomatic nodules, typically located on the lower extremities, buttocks and the scalp^[3]^. Involvement of the palms or soles is exceptionally rare^[2]^.

While SGA is usually self-limited, it may cause cosmetic concerns or discomfort, occasionally necessitating therapeutic intervention. However, surgical excision and systemic treatments are rarely indicated^[4]^.

We aim in this case to report a rarely documented palmar-dorsal hand presentation of SGA. Yet, we highlight the diagnostic significance of this uncommon benign condition and review its successful management strategy.

Review of the literature revealed that all previously reported palmar SGA nodules in healthy patients were confined exclusively to the palms. Therefore, our case represents a rare instance of simultaneous palmar-dorsal localization of SGA.

This work has been reported in line with the SCARE 2023 criteria^[5]^.

## Presentation of case

A 15-year-old female was referred to the clinic for evaluation of bilateral subcutaneous painless nodules on the dorsal surface of the proximal interphalangeal joints and palms that had been present for 5 years.

**Figure 1.**
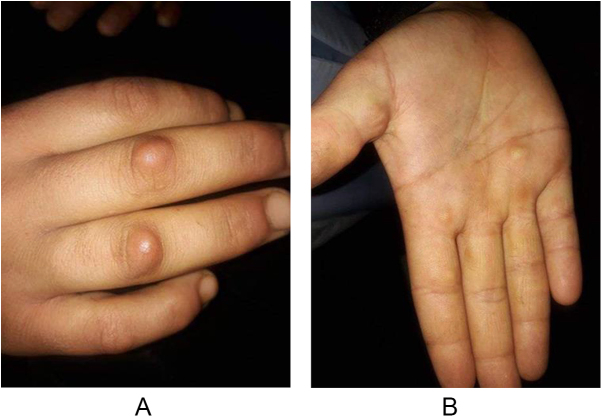
(A) SGA nodules on the dorsal surface of the proximal interphalangeal joints. (B) SGA nodules on the palms.

The patient appeared well with no significant family, past medical or smoking history. Physical examination revealed several round-to-oval, deep subcutaneous, indurated, asymptomatic, skin-colored nodules on the dorsal surface of the proximal interphalangeal joints (Fig. 1A) and palms (Fig. 1B).

There was no apparent sign of trauma and no other findings on full body skin exam including other subcutaneous nodules.

Laboratory tests, including CBC, CRP, uric acid, tuberculin test, ANA, ESR, TSH, FT4 and RF were performed and showed results within normal ranges. X-ray was normal.

**Figure 2.**
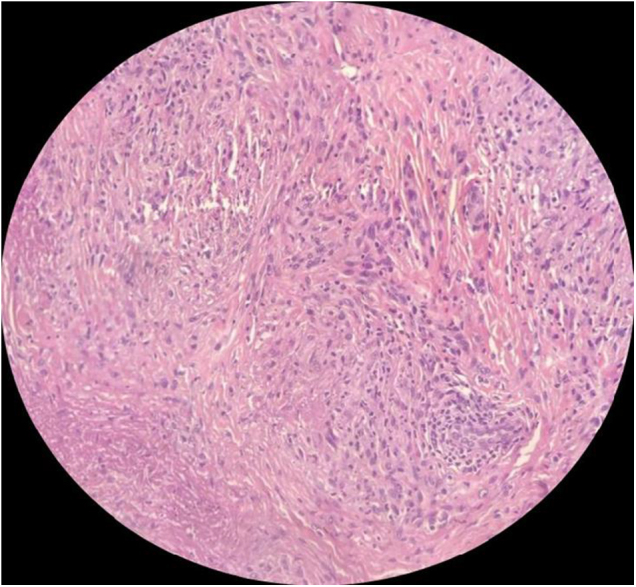
Biopsy shows numerous spindle-shaped cells surrounding the granuloma.

Excisional biopsy of the nodules revealed a palisaded granuloma with central pink fibrinous degeneration, surrounded by numerous spindle-shaped cells in the middle and deep dermis (Fig. 2). These findings are consistent with the diagnosis of SGA.

Based on the normal physical examination, unremarkable laboratory tests, and biopsy findings along with the absence of a significant medical history, the patient was diagnosed with SGA.

**Figure 3.**
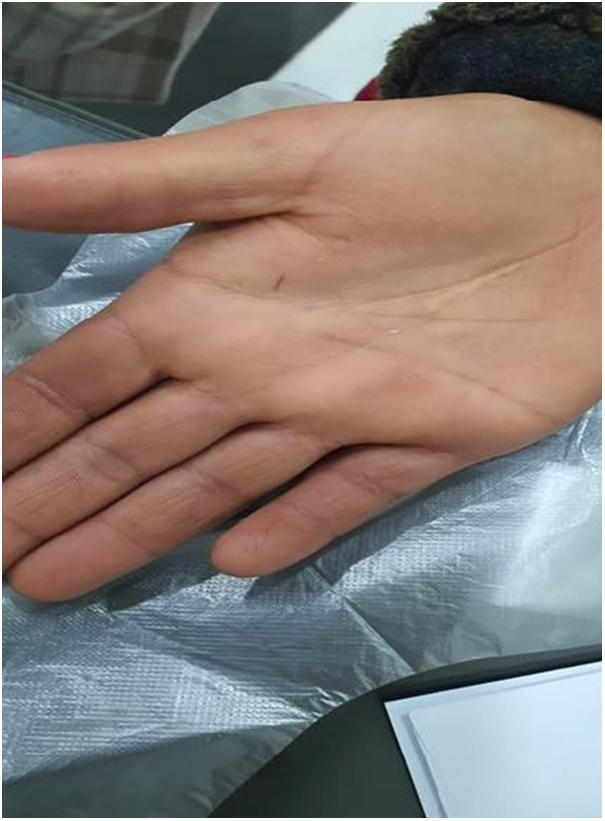
Post-treatment resolution of the lesions. Palms showing no subcutaneous nodules.

Due to cosmetic concerns, the patient was treated with intralesional triamcinolone injection. The treatment required three sessions, spaced 1 month apart between each session and we used 10 mg/ml of triamcinolone acetonide. All lesions have completely resolved with no observed side effects during or after treatment and remained clear at follow-up 8 months later (Fig. 3).

## Discussion

SGA is a rare variant of GA that predominantly affects the pediatric population^[6]^. It is an uncommon condition with an estimated prevalence of less than 2% among pediatric dermatology cases, typically presenting between 2 and 10 years of age^[1,7]^. SGA is characterized by firm, painless, non-mobile subcutaneous nodules, most frequently observed in the extremities and less commonly in the scalp or buttocks^[8]^. Histologically, SGA is defined by palisading granulomas surrounding a central necrobiotic zone with mucin deposition, a hallmark feature that distinguishes it from other similar conditions^[4].^

The etiology of SGA remains largely idiopathic, but it is hypothesized to involve a hypersensitivity reaction triggered by infections, insect bites, or trauma^[1]^.

SGA has a wide range of differential diagnoses; of these we address the subcutaneous nodules of rheumatoid arthritis that resemble SGA nodules clinically and histologically. Therefore, SGA is also called as pseudo-rheumatoid nodules^[4]^.

Rheumatoid nodules are firm, painless and mobile subcutaneous nodules located on the elbows, knees and less commonly on the dorsal surface of hands. Histologically, rheumatoid nodules are characterized by fibrous tissue containing areas of fibrinoid necrosis encircled by fibroblast and histocytes^[2,3]^. However, they were ruled out based on normal physical examination, negative serological markers and biopsy results.

**HIGHLIGHTS**

- Granuloma annulare (GA) is a benign chronic inflammatory skin disorder.
- Subcutaneous granuloma annulare (SGA) is a rare variant of GA that is seen mainly in children.
- SGA is characterized by firm, asymptomatic nodules located on the lower extremities, buttocks or scalp and rarely involves palms or soles.
- SGA is self-limited but causes discomfort and cosmetic concerns.
- Intralesional corticosteroids have been applied with variable success.

Most SGA lesions regress spontaneously, but intralesional triamcinolone or topical treatments like clobetasol have been explored with variable success^[4,7]^.

In alignment with the literature, our management strategy initially involved observation, emphasizing the self-limiting nature of SGA. However, due to the patient’s aesthetic concern and the persistence of the lesions for 5 years without resolution, intralesional triamcinolone injections were administered.

This approach aligns with previous studies advocating corticosteroid treatments for cases requiring cosmetic or functional improvement^[6,8]^.The three-session protocol proved positive, with no recurrence noted during 8 months follow-up. This highlights the potential role of intralesional corticosteroids in managing symptomatic or cosmetically significant cases.

Reported literature lacks sufficient data regarding SGA treatment. Therefore, our case could greatly contribute to future research on this topic.

What makes this case particularly significant is the bilaterality of the lesions and their localization on the palms, a presentation rarely documented in the literature.

In 2014, Na *et al*^[9]^ reported the first solitary palmar SGA nodule in a healthy child and reviewed the relevant literature. However, all reported palmar SGA nodules were located exclusively on the palms. However, our case reports simultaneous occurrence of SGA nodules on both volar and dorsal surfaces of the hand bilaterally in a healthy adolescent.

Due to the idiopathic nature of SGA, the clinical or pathophysiological significance of palmar-dorsal localization remains unclear. This uncertainty complicates diagnosis, which is already challenging due to a broad differential. However, accurate diagnosis is crucial to prevent overdiagnosis and unnecessary treatments.

## Conclusion

This case highlights a rare presentation of SGA with successful management, emphasizing its diverse clinical manifestations and diagnostic challenges. Recognizing atypical presentations is essential to avoid misdiagnosis, unnecessary surgical excision, or systemic immunosuppressants while considering patients’ aesthetic concerns.

Furthermore, this case underscores the need for longitudinal studies to assess optimal management strategies, particularly comparing long-term outcomes of corticosteroid treatment versus observation.

## Data Availability

It will be open access and publicly available as per the journal guideline.

## Research registration unique identifying number (UIN)

Since it is a case report and not a research study, no clinical trials have been performed.

## Assistance with the study

The authors sincerely thank Dr. Bakri Roumi Jamal and Dr. Fatima Breim for their invaluable assistance in enhancing this manuscript.
